# Human mesenchymal stem cells promote tumor growth via MAPK pathway and metastasis by epithelial mesenchymal transition and integrin α5 in hepatocellular carcinoma

**DOI:** 10.1038/s41419-019-1622-1

**Published:** 2019-05-29

**Authors:** Jiang Chen, Tong Ji, Di Wu, Shi Jiang, Jie Zhao, Hui Lin, Xiujun Cai

**Affiliations:** Department of General Surgery, Sir Run Run Shaw Hospital, School of Medicine, Zhejiang University, 310016 Hangzhou, Zhejiang China

**Keywords:** Cancer microenvironment, Cancer stem cells

## Abstract

Mesenchymal stem cells (MSCs) appear to be a potential vehicle for anticancer drugs due to their excellent tumor tropism ability. However, the interactions between MSCs and hepatocellular carcinoma (HCC) are quite controversial and the underlying mechanisms are ambiguous. In this study, an investigation was conducted into the effect of human MSCs (hMSCs) on tumor proliferation and metastasis both in xenograft and orthotopic models. It was discovered that hMSCs could promote tumor growth though activating mitogen-activated protein kinase (MAPK) signaling pathway and promote metastasis by epithelial mesenchymal transition (EMT) in vivo. To test whether hMSCs could induce immunosuppressive effects, the expression of the Natural killer (NK) cell marker CD56 was measured by immunohistochemical staining and the expression of interleukin-6 (IL-6) and tumor necrosis factor-alpha (TNF-α) were measured by qRT-PCR. It was found out that CD56 expression significantly decreased, while TNF-α and IL-6 expression increased in the hMSCs-treated tissues. Mechanistically, RNA sequencing was performed, which led to a discovery that integrin α5 (ITGA5) was over-expressed in hMSCs-treated HCC. ITGA5 siRNAs blocked the hMSCs-induced migration and invasion of HCC, while over-expression of ITGA5 promoted the migration and invasion ability in HCC-hMSCs, indicating that the expression of ITGA5 is associated with hMSCs-induced tumor metastasis. These findings suggest that hMSCs may play a vital role in HCC proliferation and metastasis and could be identified as a putative therapeutic target in HCC.

## Introduction

HCC is a highly aggressive tumor ranking the third leading cause of cancer death in the world^1^. Although operation techniques, novel chemo-therapies and radio-therapies are continuously improving, the prognosis of HCC remains extremely poor. Therefore, elucidating the crucial events underlying HCC tumorigenesis and exploring new therapeutic strategies to control the tumor progression is the pivotal issue to prolong patient survival^2^. Emerging evidences indicate that hMSCs can contribute to the development of tumors, demonstrating potential clinical value in cancer treatment^3–6^.

MSCs are undifferentiated cells exhibiting the capacities of self-renewal and proliferation. Under certain conditions, MSCs could be induced to differentiate into a variety of mesenchymal tissues. However, the interaction between MSCs and cancer are quite ambiguous. On the one hand, some studies showed the enhanced tumor proliferation and metastasis potential induced by MSCs^7^. On the other hand, several researches indicate that MSCs could change the stromal microenvironment and suppress the metastasis of HCC^8–11^.

Recently, several reports revealed that MSCs may play an important role in immunosuppressive effects which may contribute to cancer progression and metastasis^12,13^. It is demonstrated that MSCs could release multiple cytokines like IL6 and TNFα which exert direct effects upon cancer cells^7^. Therefore, the application of MSCs as a carrier for tumor biological therapy ought to be verified critically.

In this study, we set out to obtain further insight into the roles played by hMSCs in HCC growth and metastasis. We tried to explain why hMSCs could influence tumor growth and metastasis by measuring the proliferation and metastasis marker protein and inflammatory associated protein in tumor tissues. We also used proliferation, migration and invasion assay to investigate the role of hMSCs by co-culture system in vitro. Finally, we performed RNA sequencing to locate the differential genes between hMSCs-treated HCC and HCC alone.

## Results

### Effects of hMSCs on tumor growth and metastasis in vivo

**Fig. 1:**
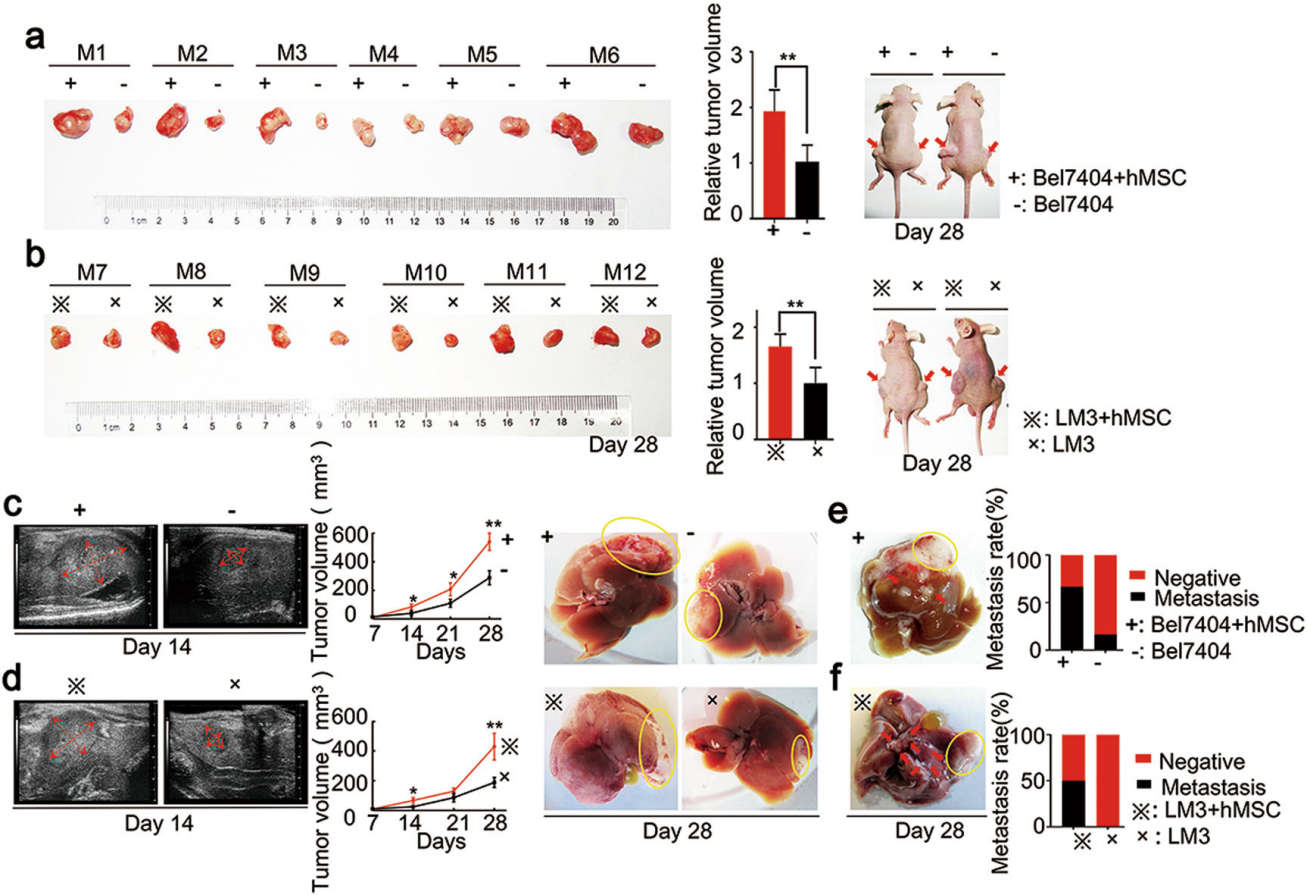
Effects of hMSCs on tumor growth and metastasis in vivo. **a** Tumor volume of Bel7404 in 6 selected pairs of xenograft model in the HCC-hMSCs group and HCC group. Photographs of tumors in 2 selected mice were showed. **b** Tumor volume of LM3 in 6 selected pairs of xenograft model in the two groups. Photographs of tumors in 2 selected mice were showed. **c** Tumor volume of Bel7404 in 6 selected pairs of orthotopic transplantation model in the hMSC-treated HCC group and HCC alone groups. **d** Tumor volume of LM3 in 6 selected pairs of orthotopic transplantation model in the two groups. **e** Photographs of metastasis in Bel7404 orthotopic transplantation model. Metastasis percentage were depicted. **f** Photographs of metastasis in LM3 orthotopic transplantation model. Metastasis percentage were depicted. B-mode ultrasound were used to quantify the relative tumor volume. mean + SEM; *P < 0.05, **P < 0.01, *N* = 6

MSCs are non-hematopoietic precursor cells and they also have been found in many human tissues, such as adipose tissue, bone marrow et al.^14,15^. MSCs, as an important ingredient in tumor microenvironment, have been confirmed to promote proliferation and metastasis in various tumors, including breast cancer, osteosarcoma, and ovarian cancer^16–18^. To identify the effect of hMSCs on the proliferation in HCC, we first started our investigation using a subcutaneous tumor model. When Bel7404/LM3 cells (HCC group) or Bel7404/LM3 cells with hMSCs (HCC-hMSCs group) were subcutaneously inoculated, tumor sizes significantly increased in HCC-hMSCs group as compared to control group (Fig. 1a, b), suggesting that hMSCs promote the tumor growth in vivo. However, it was demonstrated that Huh7/Hep3B cells with hMSCs could not promote tumor growth (Supplementary Fig. 1a, b). Next, to interrogate the role of hMSCs in tumor growth and metastasis in orthotopic model, we used Bel7404/LM3 model in situ tumor formation and found tumors turned much larger when injected HCC cells with hMSCs (Fig. 1c, d and Supplementary Fig. 1c, d). Furthermore, the metastatic nodules at liver sites indicated that hMSCs significantly promoted metastasis in vivo (Fig. 1e, f and Supplementary Fig. 1e, f). These results provide strong evidence that the hMSCs promote tumor growth and metastasis in vivo.

### hMSCs promote tumor growth though activating MAPK signaling pathway in vivo

**Fig. 2:**
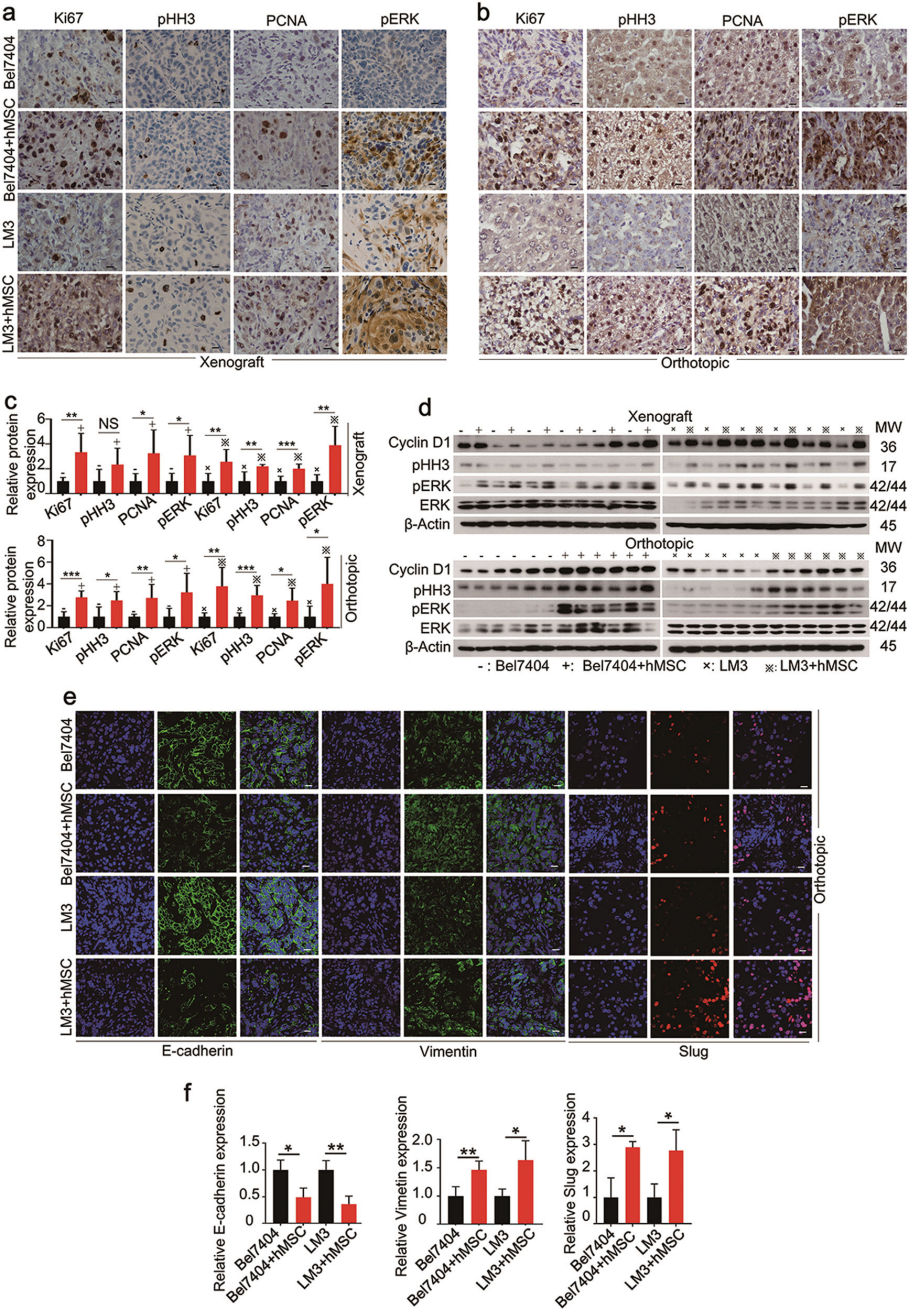
hMSCs promote tumor growth though activating MAPK signaling pathway and metastasis by EMT in vivo. **a** Representative IHC for Ki67, pHH3, PCNA, and pERK in tumors from xenograft transplantation model between the HCC-hMSCs group and HCC group. Images are at 400× magnification. **b** Representative IHC for Ki67, pHH3, PCNA, and pERK in tumors from orthotopic transplantation model in the two groups. **c** Quantification of the percentage of Ki67, pHH3, PCNA and pERK positive cells in exnograft and orthotopic transplantation models in the two groups. **d** Representative Western-bloting for Cyclin D1, pHH3 and pERK in tumors from xenograft and orthotopic transplantation models in the two groups. **e** Representative IF for E-cadherin, Vimentin and Slug in tumors from orthotopic transplantation model in the two groups. Images are at 600× magnification. **f** Quantification of the percentage of E-cadherin, Vimentin and Slug positive cells in orthotopic transplantation model in the two groups. mean + SEM; *P < 0.5, **P < 0.01, ***P < 0.001, *N* = 6

To understand how hMSCs promote HCC progression, we examined the MAPK signaling pathway, which is an important pathway for HCC growth and survival. As revealed by immunohistochemistry analysis, when compared with HCC group, HCC-hMSCs group resulted in a significant increase in the expression of phospho-p44/42 MAPK (pERK) in both xenograft and orthotopic model (Fig. 2a–c). Furthermore, to determine if MAPK signaling pathway activated in HCC-hMSCs group, we measured the expression of total extracellular signal-regulated kinase (ERK) and pERK by Western blotting assay. It was found that the level of pERK increased in HCC-hMSCs group (Fig. 2d), indicating that hMSCs promote tumor growth though activating MAPK signaling pathway.

Subsequently, we also measured the expression of proliferation-related proteins, including Ki-67, Phospho-Histone H3 (pHH3), and proliferating cell nuclear antigen (PCNA). When compared with the HCC group in both xenograft and orthotopic models, HCC-hMSCs group showed significant upregulation of protein levels of Ki-67, pHH3, and PCNA as detected by immunohistochemical and western blotting assay (Fig. 2a–d). To identify if hMSCs could affect cell cycles to promote cancer proliferation, we measured cyclin D1 level by western blotting and found the expression of cyclin D1 increased in HCC-hMSCs group (Fig. 2d). However, it was showed that there was no significantly difference in Huh7/Hep3B model (Supplementary Fig. 2a, b).

Taken together, these results demonstrate that hMSCs promote tumor growth through activating MAPK signaling pathway and increasing the expression of proliferation-related proteins, such as Ki-67, pHH3, and PCNA in vivo.

### hMSCs promote cancer metastasis by EMT in vivo

EMT has been confirmed to support cancer cells with invasive and migratory properties, promoting the initiation of metastasis^19–21^. We have discovered that hMSCs promote cancer metastasis in vivo. To test whether the metastasis by hMSCs is dependent on EMT, we measured EMT markers by immunofluorescence. In our study, we found that compared with the HCC group in Bel7404/LM3 model, HCC-hMSCs group showed significant downregulation of protein levels of E-cadherin and upregulation of protein levels of vimentin and slug (Fig. 2e, f) as detected by immunofluorescence method. These findings indicate that hMSCs promote cancer metastasis by EMT in vivo.

### hMSCs induced NK cell-suppression and TNF-α, IL-6 upregulation

Recently, MSCs have been confirmed to induce immunosuppressive properties^22,23^. NK cells are a kind of lymphocytes with innate immunity, playing a pivotal role in early host defense against cancer^24^. NK cells induce immune responses by release of cytokines, such as TNF-α, IFN-γ et al.^25^. It is illustrated that MSCs could suppress NK-cell proliferation and cytokine secretion^26^. IL-6, as an important cytokine, regulates various inflammatory factors which are responsible for inflammation, growth factors as well as angiogenic proteins which lead to tumor growth and metastasis^27^.

**Fig. 3:**
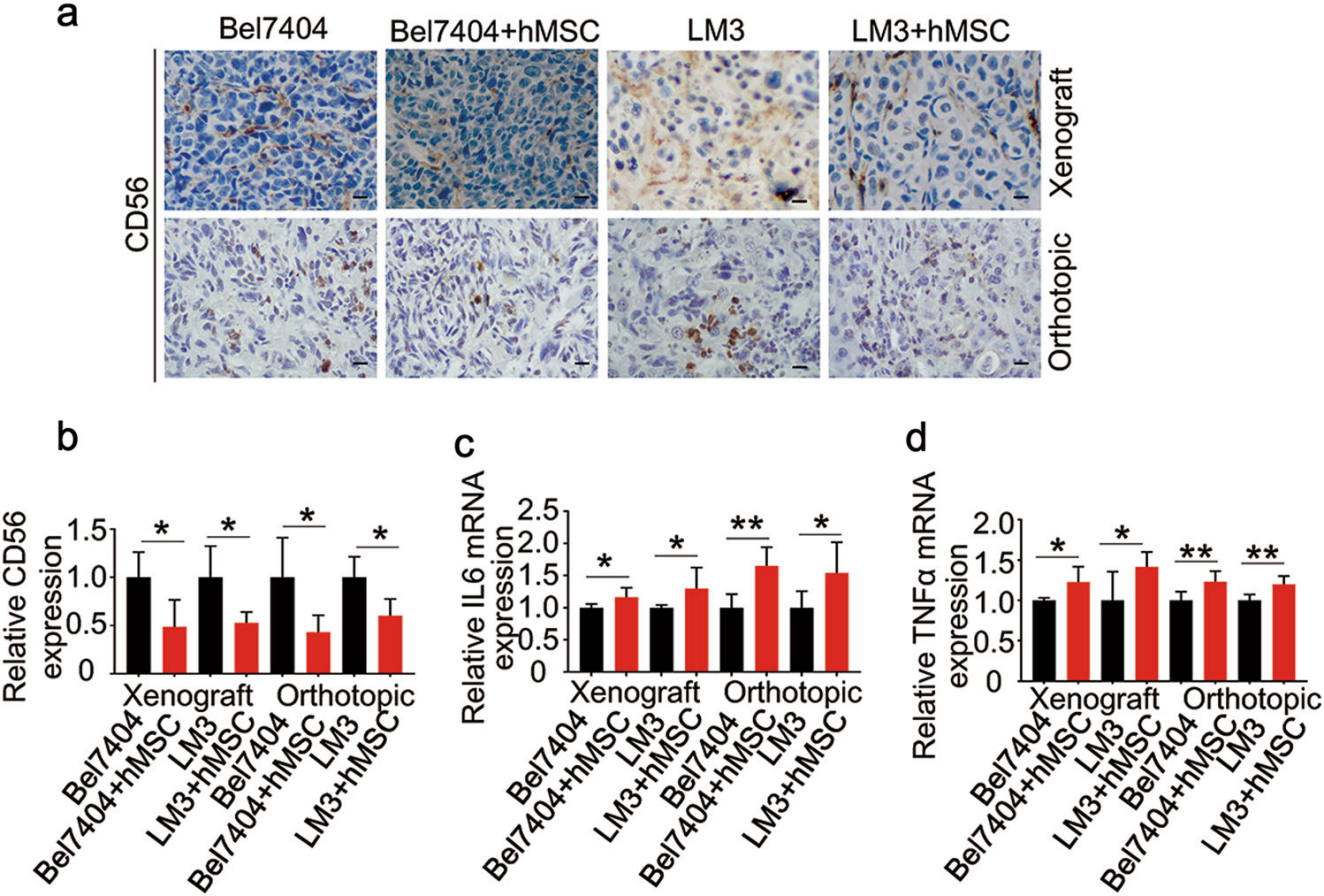
hMSCs induced NK cell–suppression and TNF-α, IL-6 upregulation. **a** Representative IHC for CD56 in tumors from Bel7404/LM3 both in xenograft and orthotopic models between the HCC-hMSCs group and HCC group. Images are at 400× magnification. **b** Quantification of the percentage of CD56 positive cells in Bel7404/LM3 both in xenograft and orthotopic models in the two groups. **c** Realtime PCR analysis of IL6 mRNA in mouse transplantation tumor in the two groups. **d** Realtime PCR analysis of TNFα mRNA in mouse transplantation tumor in the two groups. mean + SEM; *P < 0.05, **P < 0.01

In our study, compared with HCC group, the NK cell marker CD56 expression in the HCC-hMSCs group were significantly reduced by immunohistochemical staining (Fig. 3a, b). These results indicated that hMSCs inhibit NK cells and inflammation which could result in cancer progression and metastasis. To study if hMSCs could release cytokines in vivo, we measured the expression of TNF-α and IL-6 in tumor tissues by real-time PCR. The expression of TNF-α and IL-6 in the HCC-hMSCs group were significantly increased as compared to HCC group (Fig. 3c, d).

### Effects of hMSC on migration and invasion of HCC in vitro

**Fig. 4:**
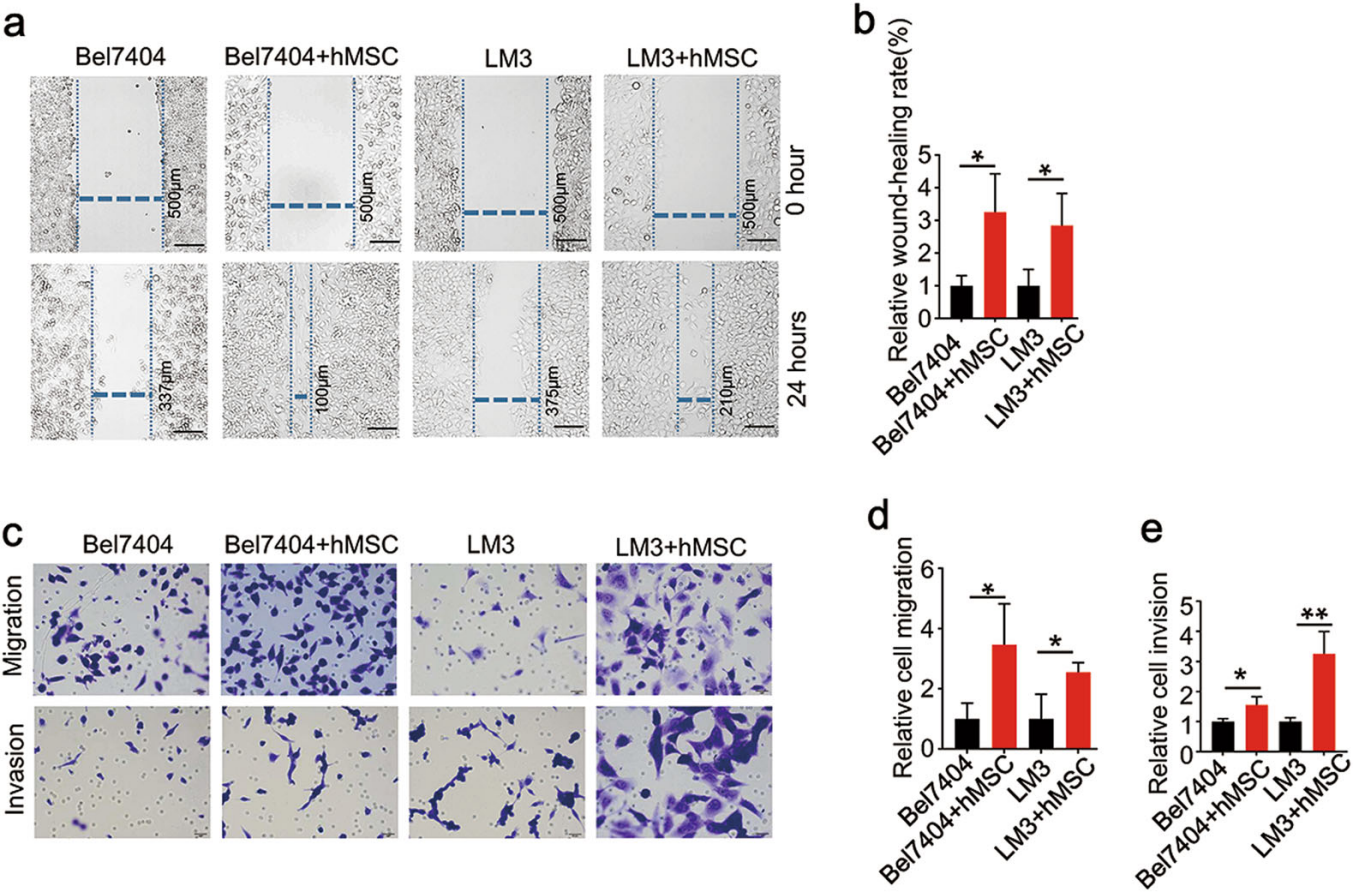
hMSCs promote migration and invasion of HCC in vitro. **a** Images of wound healing assay of Bel7404/LM3 between the HCC-hMSCs group and HCC group. Images are at 40× magnification respectively. **b** Quantification of wound healing assays of Bel7404/LM3 in the two groups. **c** Images of transwell migration and invasion assays of Bel7404/LM3 in the two groups. Images are at 400× magnification respectively. **d** Quantification of transwell migration assays of Bel7404/LM3 in the two groups. **e** Quantification of transwell invasion assays of Bel7404/LM3 in the two groups. mean + SEM; *P < 0.05, **P < 0.01

It has been confirmed that hMSCs could promote tumor growth and metastasis in several animal models. Next, we further examined the effect of hMSC in vitro. Firstly, after co-culture with hMSCs, wound-healing assay showed that the migration indexes of hMSCs-treated Bel7404/LM3 cells were significantly increased (Fig. 4a, b). The transwell migration assay also showed that the number of migrated Bel-7407/LM3 cells was much higher in HCC-hMSCs group than in HCC group (Fig. 4c, d); for the invasion assay, HCC-hMSCs group exhibited enhanced invasion ability when compared to HCC group (Fig. 4c, e). These data suggest that hMSCs promote migration and invasion of HCC cells in vitro.

Secondly, to explore if hMSCs could affect cell proliferation in HCC cell in vitro, we labeled Bel-7407/LM3 cells by Carboxyfluorescein Succinimidyl Ester (CFSE) and co-cultured with hMSCs. The results revealed that there was no difference between the two groups (Supplementary Fig. 3a, b). In our animal model, we demonstrated that cell cycle protein cyclin D1 was higher in HCC-hMSCs group (Fig. 2d). We hypothesized that the hMSCs on the growth of Bel-7407/LM3 cells may be associated with altered cell cycle progression. However, we failed to find any changes in cell cycle distribution by flow cytometric analysis (Supplementary Fig. 3c, d). Moreover, we measured PCNA, pHH3, pERK and cyclin D1 protein levels by western blotting but found no difference between the two groups (Supplementary Fig. 3e). Interestingly, these results were not consistent with the data in animal model.

Increasing evidence has showed that tumor microenvironments (TMEs), comprising extracellular matrix and cellular components, play an important role in tumor development. In addition to MSCs, fibroblasts, endothelial cells and immune cells also composed the ingredients of non-tumor cells in the TME^28^. Therefore, we suspect that other ingredients of the TME play a role in tumor growth and metastasis in vivo.

### ITGA5 in HCC is significantly upregulated by hMSCs

**Fig. 5:**
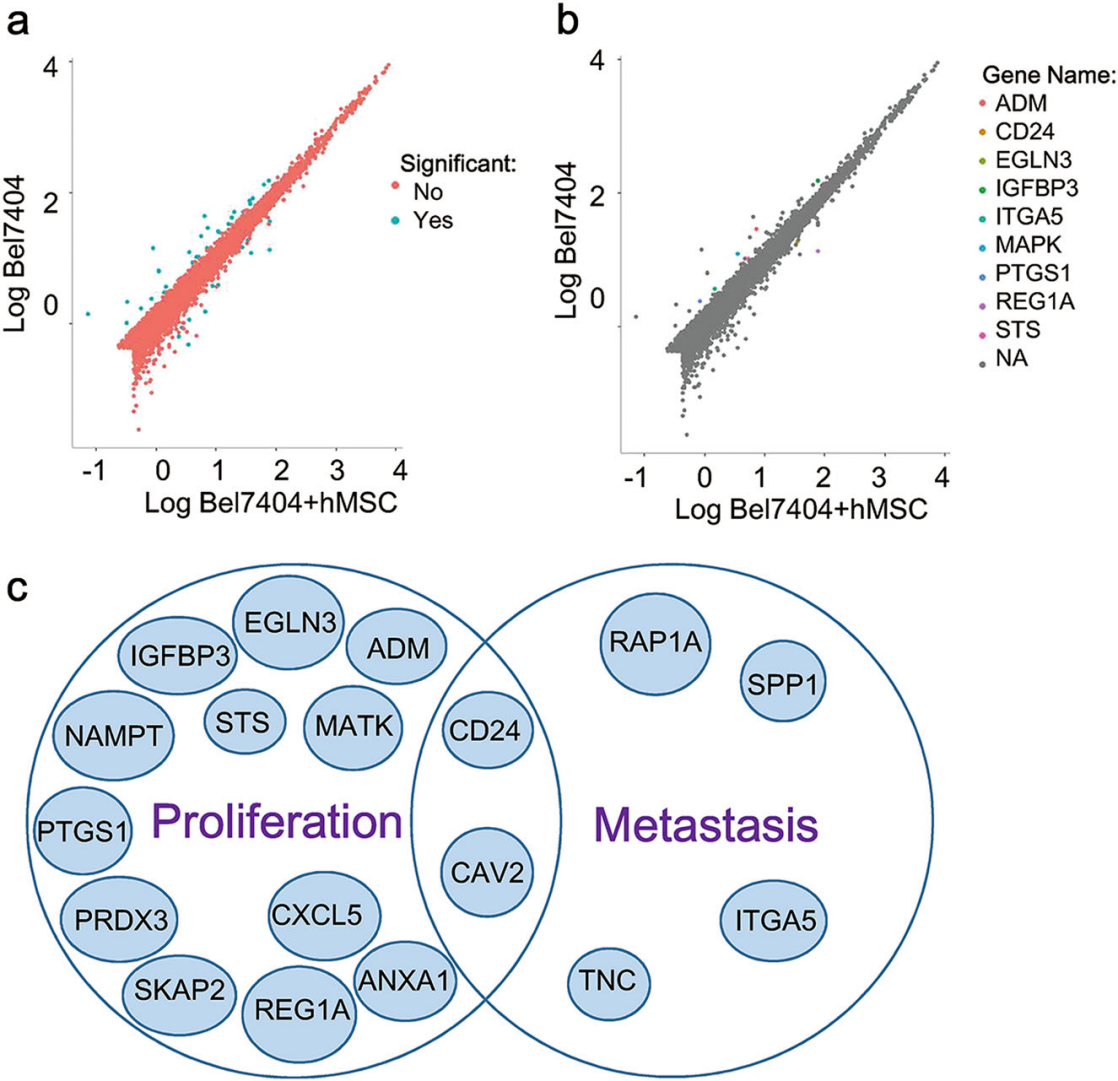
Genes in HCC are significantly changed by hMSCs. **a** RNA-seq was performed to discover gene expression changes and some significant ones were labeled with green dot. **b** Some genes with more than 2-fold change were selected from figure a and labeled with different color of dot. **c** Several genes were selected from figure a and b according to their functions such as proliferation and metastasis

To elucidate the molecular mechanism of how hMSCs promote HCC progression and metastasis, we performed RNA-sequencing to evaluate the changes in mRNAs in HCC cocultured with hMSCs (Fig. 5a–c). We identified the genes which were strongly correlated with proliferation or metastasis, and found that plenty genes were significantly changed (*P* < 0.05) (Supplementary Table 1). Subsequently, we analyzed the top most upregulated and downregulated genes (*P* < 0.05, FC log2 >1) after cultured with hMSCs in HCC and found that 14 genes correlated with proliferation and 6 genes correlated with metastasis (Fig. 5c; Supplementary Table 2).

Top-listing upregulated and downregulated genes (Supplementary Fig. 4a, b) were subjected to RNA-seq assay on tumor tissues derived from mice. Afterwards, we validated these genes in HCC group and HCC-hMSCs group by RT-qPCR assay, and discovered that the expression of ITGA5 was highly expressed in HCC-hMSCs group as compared with HCC group in vivo (Supplementary Fig. 4c, d). In addition, we further confirmed that the expression of ITGA5 was significantly higher in HCC-hMSCs group than in HCC group in vitro (Supplementary Fig. 4e, f).

**Fig. 6:**
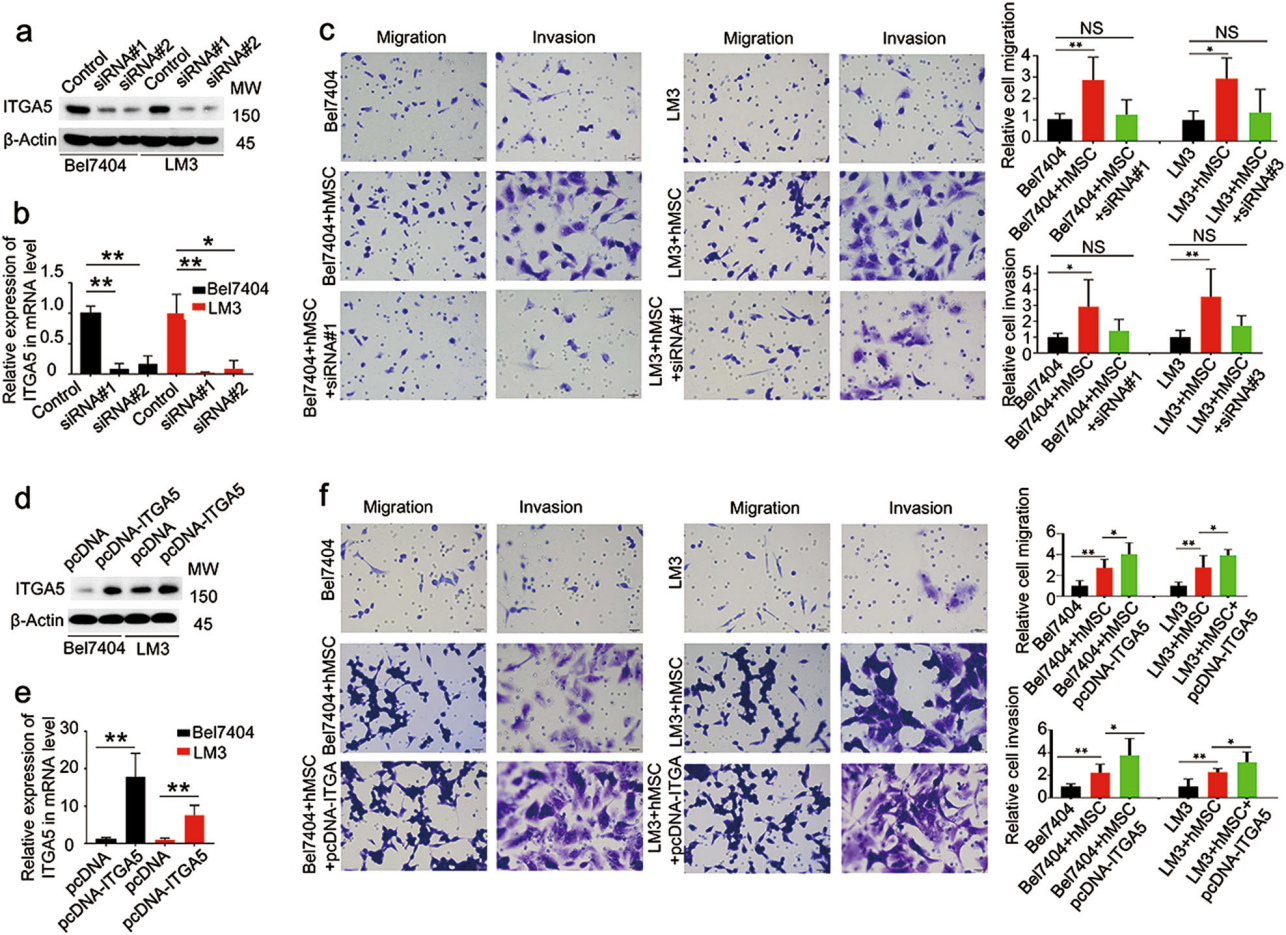
ITGA5 is correlated with hMSCs-induced migration and invasion in HCC. **a** Western blot confirming knockdown of ITGA5 following transfection with ITGA5-siRNA. **b** Realtime PCR confirming knockdown of ITGA5 following transfection with ITGA5-siRNA. **c** Images of transwell migration and invasion assays of Bel7404/LM3 following transfection with ITGA5-siRNA between HCC-hMSCs group and HCC group. Images are at 400× magnification. The quantified of transwell migration and invasion assays of Bel7404/LM3 also demonstrated. **d** Western blot confirming overexpression of ITGA5 following trans-fection with pcDNA-ITGA5. **e** Realtime PCR confirming overexpression of ITGA5 following transfection with pcDNA-ITGA5. **f** Images of transwell migration and invasion assays of Bel7404/LM3 following transfection with pcDNA-ITGA5 in the two groups. The quantified of transwell migration and invasion assays of Bel7404/LM3 also demonstrated. mean + SEM; *P < 0.05, **P < 0.01

To study the cellular functions of ITGA5, ITGA5 targeted siRNAs were used to block ITGA5 expression both in the Bel-7407/LM3 cells. Analysis with RT-qPCR and western blotting indicated that ITGA5 expression was reduced with siRNA1 and siRNA2, as compared to the control siRNA transfected cultures (Fig. 6a, b). The transwell migration and invasion assay showed that the number of migrated and invaded Bel-7407/LM3 cells was much higher in HCC-hMSCs group than in HCC group. However, treatment with ITGA5 siRNA significantly inhibited the migration and invasion ability of Bel-7407/LM3 cells of HCC-hMSCs group (Fig. 6c). Moreover, over-expressed ITGA5 both in the Bel-7407/LM3 cells and measured by RT-qPCR and western blotting (Fig. 6d, e). Our data revealed that, when compared with control groups, over-expression of ITGA5 promoted the migration and invasion ability in HCC-hMSCs (Fig. 6f). These data indicated that ITGA5 is correlated with hMSCs-induced migration and invasion in HCC cells.

**Fig. 7:**
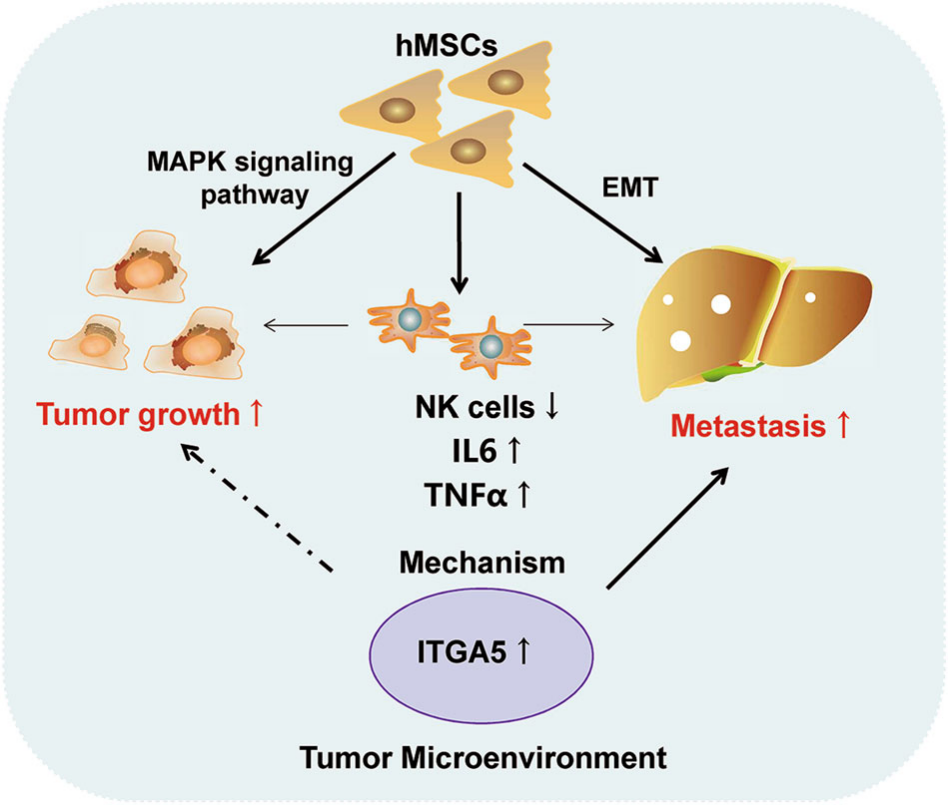
Schematic depiction. hMSCs promote tumor growth via MAPK signaling pathway and metastasis by EMT in HCC. Importantly, hMSCs induced NK cell–suppression and TNF-α, IL-6 upregulation may play an important role in tumor growth and metastasis. In addition, hMSCs promote the migration and invasion in HCC cells though targeting ITGA5

In conclusion, hMSCs could promote tumor growth though activating MAPK signaling pathway and promoting metastasis by EMT in vivo. Moreover, as compared with HCC group, CD56 expression was significantly reduced, while TNF-α and IL-6 expression was increased in the HCC-hMSCs group, which is suspected to contribute to tumor growth and metastasis. Mechanistically, we performed RNA sequencing to find out that hMSCs promote the migration and invasion in HCC though targeting ITGA5 (Fig. 7).

## Discussion

MSCs are undifferentiated cells that exhibit the capability of self-renewal and proliferation. Due to their capability to migrate as well as differentiate, MSCs play a pivotal role in the regeneration of connective tissues, preservation of tissue integrity and wound repairment^29^. Since the tumor tissue resembles similar cytokine patterns to wound healing and scar-formation, MSCs exhibit strong tropism towards tumors^8,30,31^. Therefore, MSCs could also embed into the solid tumor and contribute crucial part of the tumor microenvironment^31,32^. However, interactions between MSCs and tumor are controversial. Some researchers showed that MSCs inhibit the proliferation and metastasis of cancer cells^11,33,34^. However, others confirmed that MSCs could promote tumor growth and metastasis in different cancer types^7,8,35^. In this study, we found that hMSCs could promote tumor growth and metastasis both in Bel7404/LM3 xenograft and orthotopic models.

The activation of MAPK pathway has been shown to play a pivotal role in tumor progression^7,36–38^. In the current study, the expression of pERK was much higher in HCC-hMSCs group than in HCC group. pHH3 is a mitosis-specific marker which has already been confirmed to facilitate mitotic count in several malignancies^39,40^. Our data showed a higher expression of pHH3 in HCC-hMSCs group, demonstrating that hMSCs could promote the mitotic of cancer cells which was responsible for the promotion effect on tumor growth. However, the proliferation assay in vitro revealed that there was no difference between HCC-hMSCs group and HCC group, suggesting that the effect of hMSCs on tumorigenesis might depend on a complex tumor microenvironment.

Recently, MSCs have been confirmed to induce immunosuppressive properties^22,23^. NK cells are a kind of lymphocytes with innate immunity, showing a pivotal role in early host defense against cancer. It is showed that MSCs inhibited both the proliferation and effector functions of NK cells^41^. In order to explore the potential role played by hMSCs in immunosuppressive effect, NK cells characterized by positive expression of CD56 were detected by IHC. In our study, the expression of CD56 was lower in HCC-hMSCs group, indicating that hMSCs could inhibit NK cells and inflammation which might result in cancer progression and metastasis. Some cytokines, especially IL-6 and TNF-α, play an important role in tumorigenesis^42,43^. Also, it has been discovered that MSCs produce IL-6 which stimulates proliferation and differentiation of B cells in multiple myeloma^44^. TNF-α is one of the cytokines secreted in inflammatory processes and associated closely with tumor progression^45^. Therefore, we further measured the expression of IL-6 and TNFα in tumor tissues in mRNA level and found that the expression of IL-6 and TNF-α were higher in HCC-hMSCs group. It appears that the proliferation enhancement of tumor cells after co-culture with hMSCs depends on the growth factors, cytokines or the NK cells.

Tumor progression in the context of an altered microenvironment is characterized by increased stromal-epithelial interactions, altered integrins expression and extracellular matrix disorder^46,47^. Functional properties of integrins are versatile, for which they could provide traction for cell migration and assemble the extracellular matrix by transmitting signals out of the cell^48^. Some of integrin family, with integrin-α in particular, has been demonstrated to be altered in certain cancers such as esophageal carcinoma, gastric cancer, breast cancer and non-small cell cancer^47,49–52^. Recent researches have revealed that over-expression of ITGA5 in cancer cells induced improved invasion ability and epithelial to mesenchymal transition^47^. Here, we revealed higher expression of ITGA5 in hMSCs-treated HCC cells which exhibited enhanced migration and invasion ability. More importantly, the cell motility improvement was inhibited after knocking down the expression of ITGA5 in HCC cells, indicating an important role of ITGA5 in the progression of HCC. The precise mechanism elucidating this phenomenon needs further study.

However, there were several limitations in this study. Firstly, in the study, the cultivation of HCC cells with hMSCs resulted in higher proliferation in vivo but not in vitro model. As reported, hMSCs could be induced to differentiate into a variety of mesenchymal cells under different differentiation environment. But unfortunately, in this study, there was no robust evidence to demonstrate that certain surface marker protein. In our view, TMEs are very complex and some other ingredients of the TME may play a role in tumor growth and metastasis in vivo. Secondly, some articles showed that MSCs could mediate macrophage polarization and function^53^. However, in our study, the number of macrophages in HCC-hMSCs group is lower than HCC group (data not shown). Thirdly, in this study, several HCC cell lines were used to draw the conclusion. As is mentioned above, different cell lines have different response to hMSC coculture. That’s why there is no unanimous opinion about the role of hMSC in HCC.

In conclusion, our study indicates a possibility illustrating the effects of hMSCs on the proliferation and metastasis of HCC. Firstly, we found that hMSCs promote tumor growth and metastasis on HCC in vitro and in vivo. Furthermore, we revealed that hMSCs promote tumor growth by activating of MAPK signaling pathway, and facilitate metastasis via mediating EMT in vivo. In addition, we also confirmed that hMSCs could exert immunosuppressive effects, specifically, NK cell-suppression and TNF-α, IL-6 upregulation. Mechanistically, the differential genes between HCC-hMSCs group and HCC group were discovered by RNA sequencing assay. To confirm which gene resulted in the effects of hMSCs, we performed RT-PCR and considered that upregulation of ITGA5 may play a pivotal role in migration and invasion of HCC. Targeting hMSCs could represent a new strategy to control the progression of HCC.

## Materials and methods

### Cell lines and culture conditions

The hMSCs cell line, which was isolated from human bone marrow and characterized by immunofluorescent methods, was purchased from American Type Culture Collection (ATCC®PCS-500-012™, Manassas, VA, USA). The cells were cultured in modified Eagle’s medium (MEM) (Gibco Invitrogen, Karlsruhe, Germany) containing 7% fetal bovine serum (FBS) (HyClone, Logan, UT), with 10 Units/mL penicillin, 10 µg/mL streptomycin, 25 ng/mL amphotericin B (ATCC PCS999002, Manassas, VA, USA), 15 ng/mL rh IGF-1, 5 ng/mL Rh FGF-b and 2.4 mM l-Alanyl-l-Glutamine (ATCC PCS-500-04, Manassas, VA, USA) in 5% CO_2_ at 37 °C. The human HCC cell line LM3, Huh7 and Hep3B were obtained from ATCC in 2010. The Bel7404 cell line was a gift from Wu Xi App Tec Co. Ltd. Bel7407, Huh7, LM3 and Hep3B were cultured in DMEM containing 10% FBS and 1% streptomycin/penicillin in 5% CO_2_ at 37 °C.

### In vivo tumorigenesis assays

In the xenograft model, 4–6 weeks mice were divided into 4 groups (each with 6 mice). A total 1 × 10^6^ Bel7407 cells or 1 × 10^6^ LM3 cells or 1 × 10^6^ Huh 7 cells or 1 × 10^6^ Hep3B cells were injected subcutaneously into the right flank and a mixture of equal numbers of hMSCs (1 × 10^5^) plus 1 × 10^6^ Bel7407 cells or 1 × 10^6^ LM3 cells or 1 × 10^6^ Huh 7 cells or 1 × 10^6^ Hep3B cells (1 × 10^6^) cells were injected subcutaneously into the left flank. All mice were kept in pathogen-free conditions. At 4 weeks post-injection, mice were sacrificed and tumors were harvested and measured. A portion of each tumor tissue was fixed in 4% formaldehyde for immunohistochemical analysis and others were stored in liquid nitrogen for protein and mRNA analysis.

In the Orthotopic model, 4–6 weeks mice were divided into 8 groups (each with 6 mice), Groups 1–4 consisted of mice that were inoculated in the left lobe of liver with a mixture of equal numbers of hMSCs (1 × 10^5^) plus Bel7407 (1 × 10^6^) cells or LM3 (1 × 10^6^) cells or Huh7 (1 × 10^6^) cells or Hep3B (1 × 10^6^) cells respectively. Groups 5–8 consisted of mice inoculated in the left lobe of liver with 1 × 10^6^ LM3 cells or 1 × 10^6^ Huh 7 cells or 1 × 10^6^ Hep3B cells only, respectively. All mice were kept in pathogen-free conditions. Tumor size was measured twice a week using B-mode ultrasound and the volume of tumors was calculated as *V* = (length × width^2^)/2. At 4 weeks post-injection, mice were sacrificed and tumors were harvested. A portion of each tumor tissue was fixed in 4% formaldehyde for immunohistochemical analysis and others were stored in liquid nitrogen for protein and mRNA analysis.

### Immunohistochemistry (IHC)

IHC were performed to examine cell proliferation marker as Ki67, pHH3, PCNA, and pERK in tumor tissues. IHC were also performed to examine inflammatory cell marker CD56. After being processed for paraffin embedding, 5 μm sections of tissue samples were prepared. Sections were boiled in 10 mM sodium citrate buffer (PH 6.0) for 20 min, and incubated in 0.3% hydrogen peroxide for 20 min and then blocked with 5% BSA for 1 h. Then incubated anti-Ki67 (Cell Signaling Technology, IHC, 1:400), anti-Phospho-Histone H3(Cell Signaling Technology, IHC, 1:50), anti-PCNA (Cell Signaling Technology, IHC, 1:4000) and anti-Phospho-p44/42 MAPK (Erk1/2) (Thr202/Tyr204) (Cell Signaling Technology, IHC,1:400), anti-CD56(Cell Signaling Technology, IHC, 1:800) antibodies overnight at 4 °C, followed by biotinylated secondary antibodies and DAB detection.

### immunofluorescence (IF)

IF were performed to examine migration and invasion associated genes such as E-cadherin, Vimentin and Slug in tumor tissues. Sections were boiled in 10 mM sodium citrate buffer (PH 6.0) for 20 min, and blocked with 5% BSA for 1 h. Sections were incubated overnight at 4 °C with E-cadherin (Cell Signaling Technology, 1:50), Vimentin (Cell Signaling Technology, 1:100), Slug (Cell Signaling Technology, 1:400) primary antibodies. After being washed, sections were incubated with anti-rabbit IgG secondary antibodies (Cell Signaling Technology, 1:200). Counter stained with DAPI stain.

Western blot analysis: Total protein was extracted from cells and tumor tissues. The protein concentration was determined by BCA assay (Therom Fisher Scientific, USA). Equal amounts of protein were loaded into each lane, separated by 10% SDS-PAGE, electrotransferred to the PVDF membrane. After incubating in blocking buffer for 1 h at room temperature, the membrane were incubated with primary antibody against anti-Phospho-p44/42 MAPK (Erk1/2) (Thr202/Tyr204) (Cell Signaling Technology, WB, 1:2000), anti-p44/42 MAPK (Erk1/2) (Cell Signaling Technology, WB, 1:1000), anti-β-Actin (Cell Signaling Technology, WB, 1:1000), anti-phospho-Histone H3(Cell Signaling Technology, WB, 1:1000), anti-PCNA(Cell Signaling Technology, WB, 1:2000), anti-ITGA5(Cell Signaling Technology, WB, 1:1000), anti-Cyclin D1 (Cell Signaling Technology, WB, 1:1000) overnight at 4 °C. Then the membrane was incubated with HRP-conjugated secondary antibody for 2 h at room temperature.

### Quantitative real-time PCR

Total RNA was extracted from cells or animal model tissues using Trizol (Invitrogen, Carlsbad, CA, USA). Reverse transcription was performed by PrimeScript RT reagent kit (Takara, Dalian, China). qPCR was performed using SYBR premix Ex Taq (Bio-Rad, Hercules, CA, USA). Gene expression in samples was normalized by house-keeping gene expression. Relative quantification of target gene expression was evaluated using the comparative CT method. Sequences of all primers are listed (Supplementary Table 3).

### Cell proliferation assay

To determine the effect of hMSCs on HCC cells in vitro, CFSE dye (Life Technologies-Molecular Probes, Grand Island, NY, USA) was used to detect the proliferation capacity. Bel7404/LM3 cells were incubated with 10 nM CFSE for 30 min and washed with complete medium, prior to being mixed with hMSCs in the co-culture system. A number of hMSCs (1 × 10^4^) with Bel7404/LM3 cells (1 × 10^5^) or Bel7404/LM3 cells (1 × 10^5^) alone were cultured for 36 h. The CFSE^+^ cells in the inserts were counted in triplicate under a microscope after incubation for 36 h.

### Flow cytometric analysis

A 6-well plate and 3 μm pore size transwell inserts (Corning) were used to establish the co-culture systems as reported before^54^. Bel7404/LM3 cell lines were seeded in a prepared 6-well plate with a number of 5 × 10^4^ cells/well. A number of 5 × 10^4^ cells/well of hMSCs were seeded in the transwell inserts located in neighboring wells. After cells were attached to the wall firmly, the transwell inserts with hMSCs were moved to the wells containing Bel7404/LM3. The Bel7404/LM3 in this coculture system was regarded as co-culture groups. In control groups, both plate wells and transwell inserts were seeded with Bel7404/LM3. After incubation for 48 h, the co-cultured cells (Bel7404/LM3 cells) were harvested. After being washed with PBS, cells were fixed with 70% ice-cold ethanol, incubated with Cell Cycle Staining Kit (BD Biosciences, San Jose, CA, USA) for 30 min in the dark, and analyzed by flow cytometry.

### Cell migration and invasion assay

For cell migration assay, the 1 × 10^6^ HCC cells were cultured in FBS-free DMEM for 24 h, and then 2 × 10^5^ cells were re-suspended in DMEM and seeded in the upper well of the transwell chamber (BD Biosciences, Bedford, MA, USA). The lower wells were added 2 × 10^5^ cells of hMSCs in 600 μL DMEM containing 10% FBS as the chemoattractant. After incubation at 37 °C for 48 h, the cells in the upper surfaces of the filter were removed with cotton swabs, and cells that had migrated onto the lower surfaces of the filter were fixed with 4% formaldehyde for 20 min and then stained with Geimsa and counted under a microscope at ×400 magnification. For cell invasion assay, 80 μl serum-free DMEM-diluted Matrigel (BD, San Jose, CA, USA) was added to the transwell filter and incubated at 37 °C for 2 h to form a matrix gel. The incubation time would be prolonged to 72 h. The lower wells were added 2 × 10^5^ cells of hMSCs in 600 μl DMEM supplemented with 10% FBS as the chemoattractant. After incubation for 72 h, the cells in the upper surfaces of the filter were removed with cotton swabs, and cells that had migrated onto the lower surfaces of the filter were fixed with 4% formaldehyde for 20 min and then stained with Geimsa for counting under a microscope at ×400 magnification.

### Wound healing assay

Bel7404/LM3 cells alone or Bel7404/LM3 cells with hMSCs were grown to 70% confluency. Scratch was done by a sterile 10 µL pipette tip and photomicrographs were taken with 40× microscope (Olympus) at 0 and 24 h. For each time point, 5–10 photomicrographs were taken from three parallel wells. The mean area of the cell deprived scratch zone was measured and compared to the 0-hour time point by using ImageJ software.

### RNA sequencing analysis

RNA isolation for RNA sequencing (RNA-seq) analyses was conducted using TRIzol (Invitrogen, Carlsbad, CA, USA). The RNA-seq analysis for Bel7404 with hMSC or Bel7404 cells alone were performed using Illumina HiSeq2500 system (Illumina, San Diego, CA). The reads were aligned against the human reference genome (hg19) and TMM normalization method was applied for data normalization (R/Bioconductor package edgeR). R language software was used for gene expression visualization and to generate heatmaps.

### siRNA and plasmid construction and cell transfection

Bel7404/LM3 cells were cultured to 50% confluence and transfected with following siRNAs: 5′-(GCAAGAAUCUCAACAACUCUU)d(TT)-3′(ITGA5 siRNA1), 5′-(GAGAGGAGCCUGUGGAGUAUU)d(TT)-3′(ITGA5 siRNA2) targeting two distinct areas in ITGA5 (Dharmacon Research, Lafayette, CO). Control: 5′-(GGCUCCCGCUGAAUUGGAAUU)d(TT)-3′. The plasmids were constructed by restriction‐enzyme double digestion and ligation. Transfections were performed using Oligo fectamine (Invitrogen) according to the manufacturer’s instructions.

### Statistical analysis

The data were analyzed using SPSS statistical analysis software Version17.0. Student’s *t*-test was performed to examine the statistical significance when two groups were compared. One-way ANOVA was used to analyze the differences among three or more groups. *P*-value < 0.05 was regarded as statistically significant.

## Supplementary information

Supplement Figure S1

Supplement Figure S2

Supplement Figure S3

Supplement Figure S4

Table S1

Table S2

Table S3

Supplementary figure legends
