# Author Correction: Human mesenchymal stem cells promote tumor growth via MAPK pathway and metastasis by epithelial mesenchymal transition and integrin α5 in hepatocellular carcinoma

**DOI:** 10.1038/s41419-019-1721-z

**Published:** 2019-06-20

**Authors:** Jiang Chen, Tong Ji, Di Wu, Shi Jiang, Jie Zhao, Hui Lin, Xiujun Cai

**Affiliations:** Department of General Surgery, Sir Run Run Shaw Hospital, School of Medicine, Zhejiang University, 310016 Hangzhou, Zhejiang China

**Keywords:** Cancer microenvironment, Cancer stem cells

**Correction to: Cell Death and Disease**

10.1038/s41419-019-1622-1 published online 29 May 2019

Since publication of this article, the authors noticed that the author list had been changed from “Jiang Chen^1^, Tong Ji^1^, Di Wu^1^, Shi Jiang^1^, Jie Zhao^1^, Hui Lin^1^ and Xiujun Cai^1^” to “Jiang Chen^1^, Tong Ji^1^, Di Wu^1^, Shi Jiang^1^, Jie Zhao^1^, Xiujun Cai1 and Hui Lin1”

The correct list should be “Jiang Chen^1^, Tong Ji 1, Di Wu^1^, Shi Jiang^1^, Jie Zhao^1^, Hui Lin^1^ and Xiujun Cai^1^”,

This has been corrected in both the PDF and HTML versions of the Article.

