# Supplementary figures and images for: Human mesenchymal stem cells promote tumor growth via MAPK pathway and metastasis by epithelial mesenchymal transition and integrin α5 in hepatocellular carcinoma
Source: Cell Death Dis. 2019 May 29;10(6):425. doi: 10.1038/s41419-019-1622-1 (PMC6541606; doi:10.1038/s41419-019-1622-1)

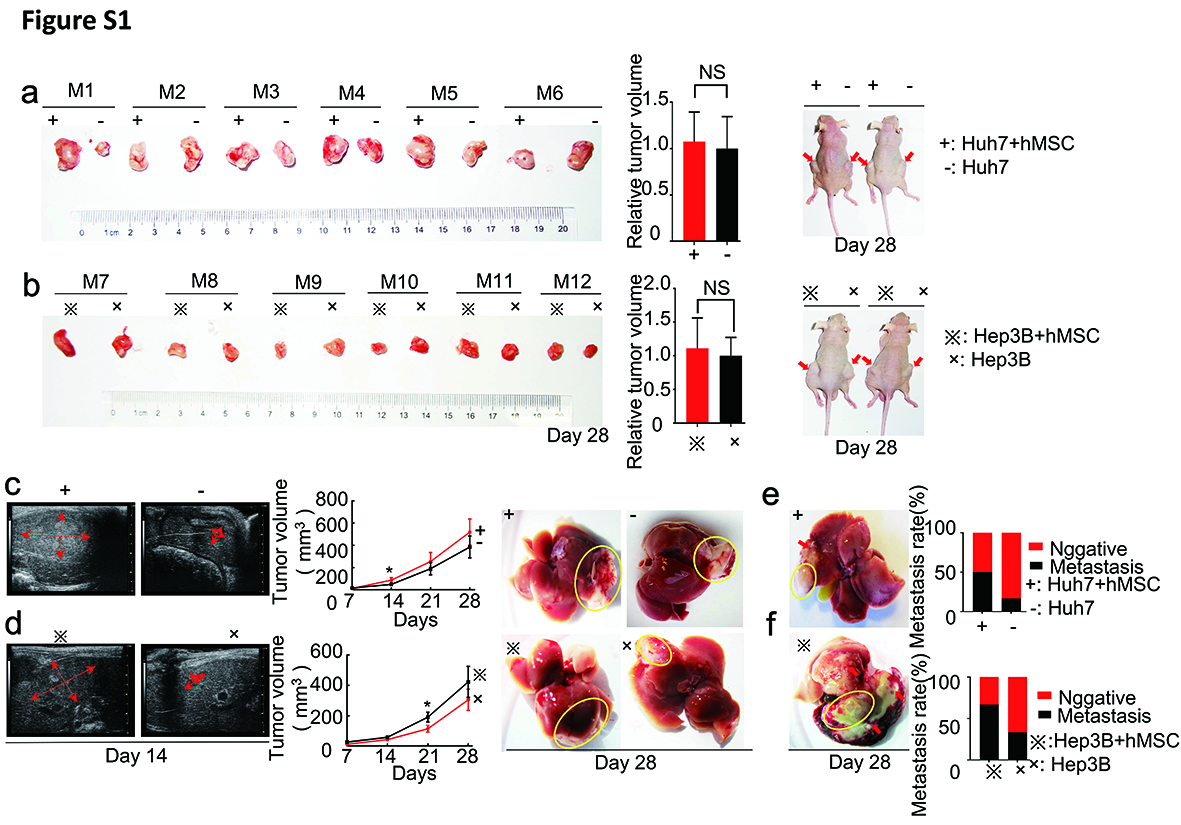

Supplement: Supplementary file 1 — Supplement Figure S1 [file 41419_2019_1622_MOESM1_ESM.tif]

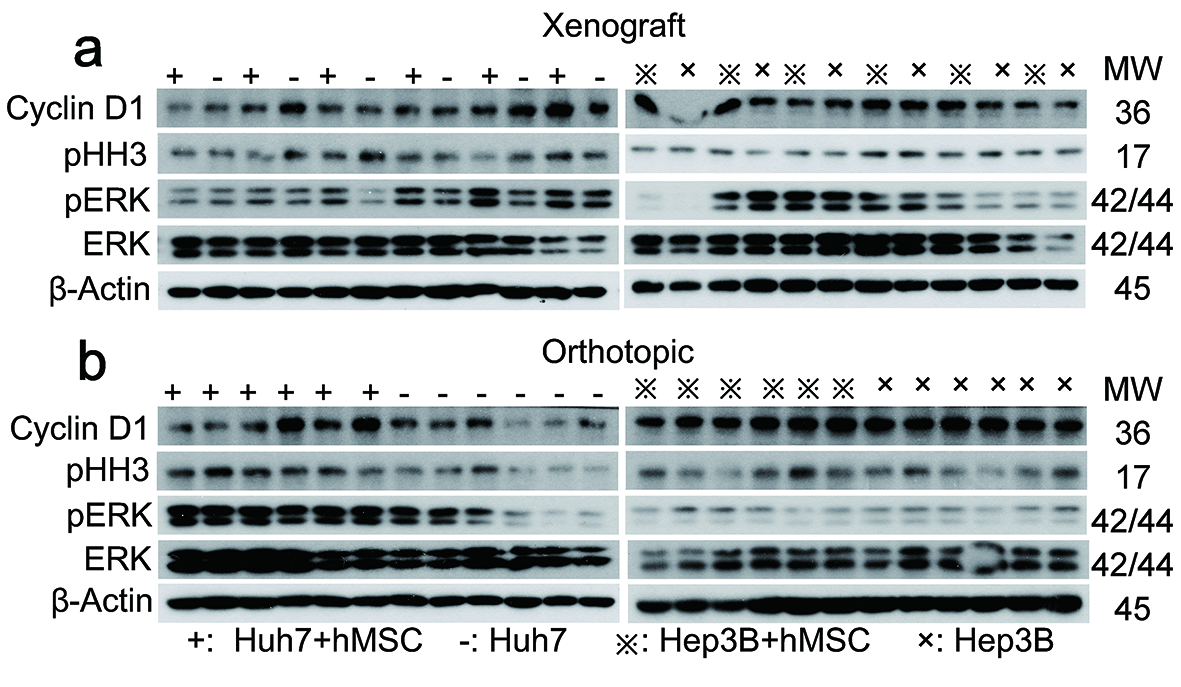

Supplement: Supplementary file 2 — Supplement Figure S2 [file 41419_2019_1622_MOESM2_ESM.tif]

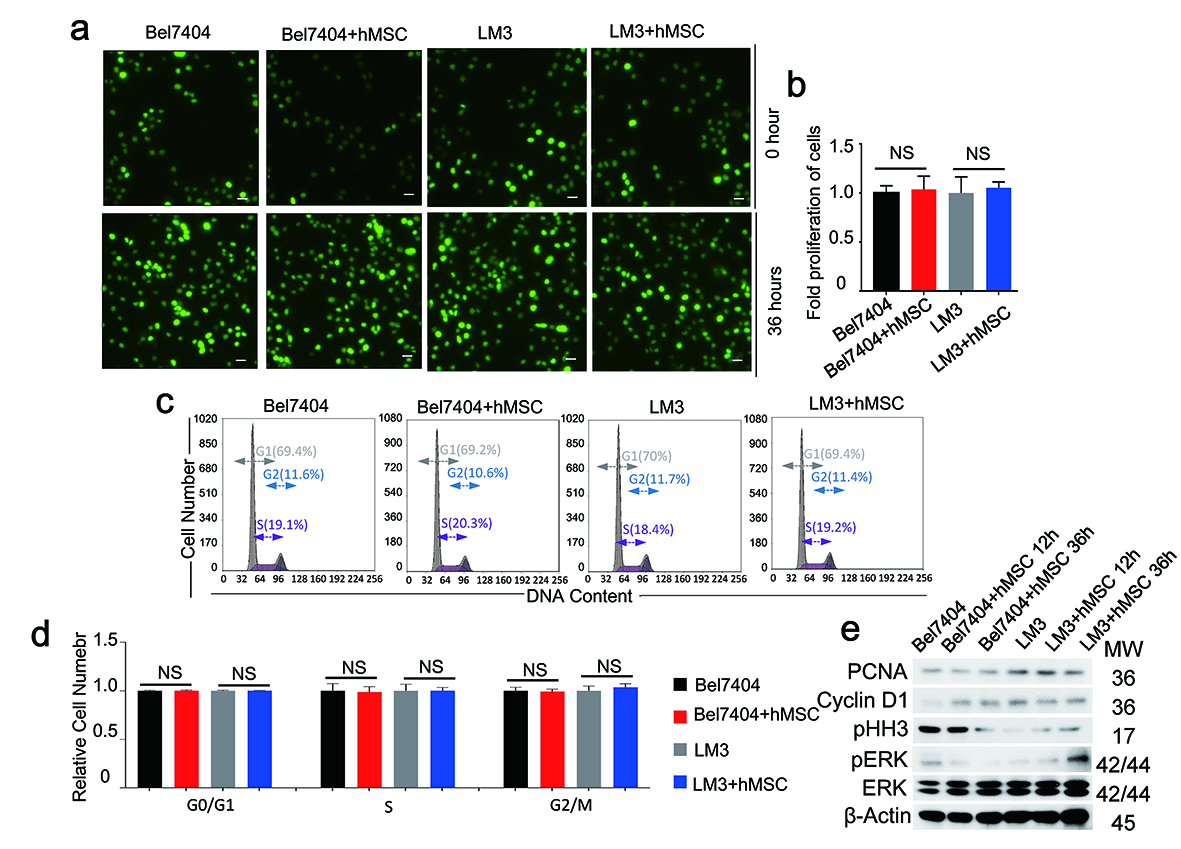

Supplement: Supplementary file 3 — Supplement Figure S3 [file 41419_2019_1622_MOESM3_ESM.tif]

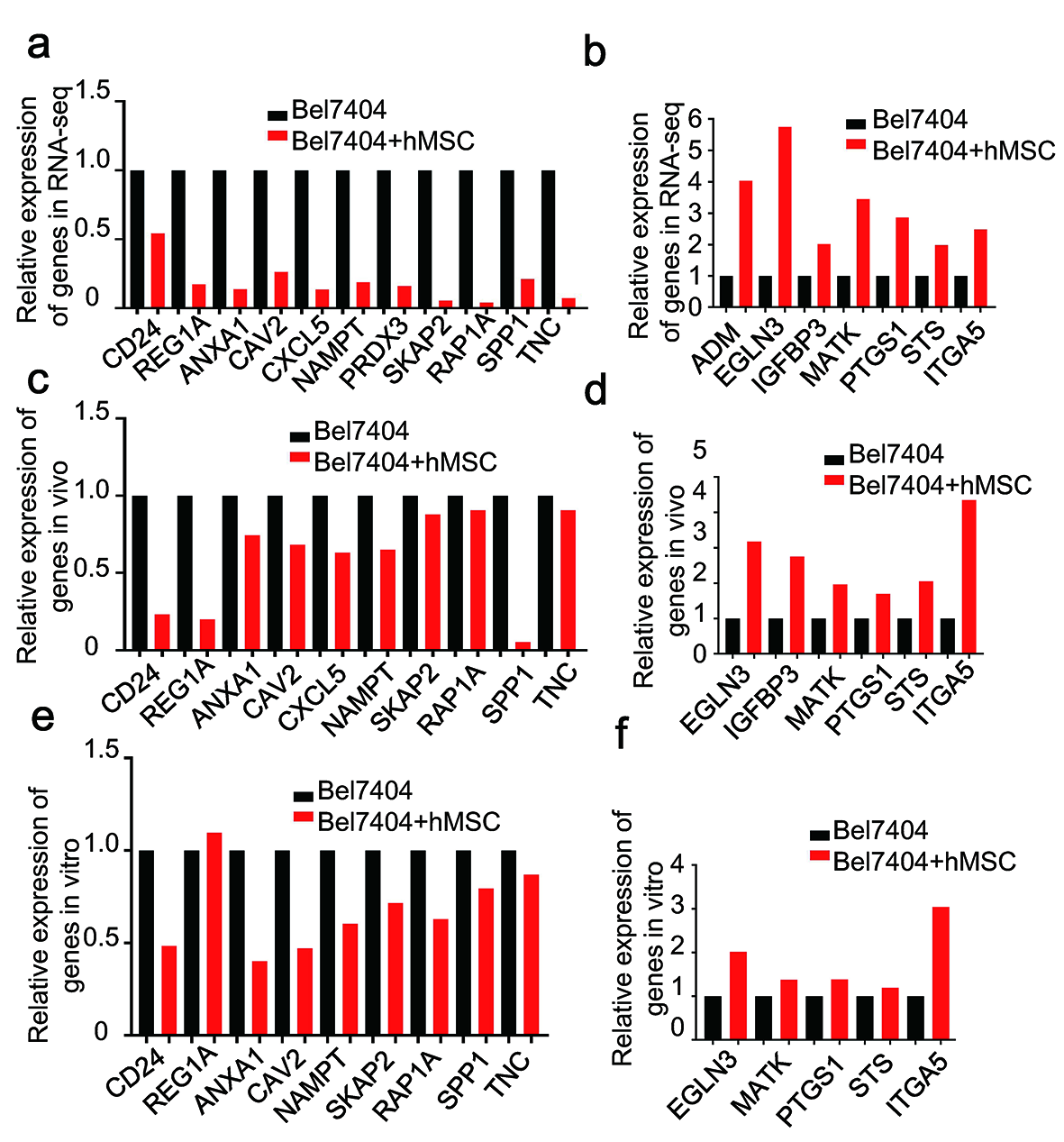

Supplement: Supplementary file 4 — Supplement Figure S4 [file 41419_2019_1622_MOESM4_ESM.tif]
