# Supplementary material for: Human mesenchymal stem cells promote tumor growth via MAPK pathway and metastasis by epithelial mesenchymal transition and integrin α5 in hepatocellular carcinoma
Source: Cell Death Dis. 2019 May 29;10(6):425. doi: 10.1038/s41419-019-1622-1 (PMC6541606; doi:10.1038/s41419-019-1622-1)
Supplement: Supplementary file 7 — Table S3 [file 41419_2019_1622_MOESM7_ESM.docx]

Table S2.

**Oligonucleotide primers.**

| Primer | Sequence (5’ to 3’) | Location |
| --- | --- | --- |
| IL6 | TCCAGTTGCCTTCTTGGGAC | sense |
| IL6 | GTGTAATTAAGCCTCCGACTTG | antisense |
| TNFα | CAGGAGGGAGAACAGAAACTCCA | sense |
| TNFα | CCTGGTTGGCTGCTTGCTT | antisense |
| CD24 | CAGGGCAATGATGAATGAGAAT | sense |
| CD24 | CCTGGGCGACAAAGTGAGA | antisense |
| REG1A | AGGAGAGTGGCACTGATGACTT | sense |
| REG1A | TAGGAGACCAGGGACCCACTG | antisense |
| ANXA1 | CTAAGCGAAACAATGCACAGC | sense |
| ANXA1 | CCTCCTCAAGGTGACCTGTAA | antisense |
| CAV2 | CACCCTCAGCTGTCTGCACAT | sense |
| CAV2 | GGCAGAACCATTAGGCAGGTCTT | antisense |
| CXCL5 | GCTGGTCCTGCCGCTGCTGTG | sense |
| CXCL5 | GTTTTCCTTGTTTCCACCGTC | antisense |
| NAMPT | CAGGATGATGAGGACAGCACC | sense |
| NAMPT | CTCTGCAGACTCAAACTCCAC | antisense |
| PRDX3 | ACTGTGAAGTTGTCGCAGTCT | sense |
| PRDX3 | CACACCGTAGTCTCGGGAAA | antisense |
| SKAP2 | GAGGTGCTCCCAGAGGATGACA | sense |
| SKAP2 | CAGTCCCACAAGCCCTGGTAGT | antisense |
| RAP1A | GAAGAACGGCCAAGGTTTTGC | sense |
| RAP1A | CCGTGTCCTTAACCCGTAAAATC | antisense |
| SPP1 | TGGGAATAGCTTTGGGAAGTGG | sense |
| SPP1 | CCGATGTCCAAAGGTGCAAT | antisense |
| TNC | AGGGCAAGTGCGTAAATGGAG | sense |
| TNC | TGGGCAGATTTCACGGCTG | antisense |
| ADM | TAAGTGGGCGCTAAGTCGTG | sense |
| ADM | TCTCATCAGCGAGTCCCGTA | antisense |
| EGLN3 | AGAAAGGGCAGAAGCCAAAAAG | sense |
| EGLN3 | GCGGATTGCACACCACAGT | antisense |
| IGFBP3 | AAGACAGCCAGCGCTACAAAG | sense |
| IGFBP3 | TACGGCAGGGACCATATTCTG | antisense |
| MATK | CACCAAGCTGGTGAAGCCAAGGAG | sense |
| MATK | AGAGCAAACTGCAGA AGCTGAGAG | antisense |
| PTGS1 | TGTGGATGTCATCAGGGAGTCT | sense |
| PTGS1 | GAAGGAGGTGTAGGGCTTCATG | antisense |
| STS | AGGGTCTGGGTGTGTCTGTC | sense |
| STS | ACTGCAACGCCTACTTAAATG | antisense |
| ITGA5 | TCATCTACATCCTCTACAAGCTTGG | sense |
| ITGA5 | GCCGTCAGCACCTTCAAGA | antisense |
| GAPDH | GTCAGCCGCATCTTCTTTTG | sense |
| GAPDH | GCGCCCAATACGACCAAATC | antisense |
