# Supplementary material for: Human mesenchymal stem cells promote tumor growth via MAPK pathway and metastasis by epithelial mesenchymal transition and integrin α5 in hepatocellular carcinoma
Source: Cell Death Dis. 2019 May 29;10(6):425. doi: 10.1038/s41419-019-1622-1 (PMC6541606; doi:10.1038/s41419-019-1622-1)
Supplement: Supplementary file 8 — Supplementary figure legends [file 41419_2019_1622_MOESM8_ESM.docx]

**Figure S1.** **Effects of hMSCs on tumor growth and metastasis in vivo**.

(a) Tumor volume of Huh7 in 6 selected pairs of xenograft model in the HCC-hMSCs group and HCC group. Qunantification of relative tumor volumes were demonstrated. (b) Tumor volume of Hep3B in 6 selected pairs of xenograft model in the two groups. Qunantification of relative tumor volumes were demonstrated. (c) Tumor volume of Huh7 in 6 selected pairs of orthotopic transplantation model in the two groups. (d) Tumor volume of hep3B in 6 selected pairs of orthotopic transplantation model in the two groups. (e) Photographs of metastasis in Huh7 orthotopic transplantation model. Metastasis percentage were depicted. (f) Photographs of metastasis in Hep3B orthotopic transplantation model. Metastasis percentage were depicted. B-mode ultrasound were used to quantify the relative tumor volumes. Photographs of tumors in 2 selected mice were showed. mean + SEM; **P*<0.05, NS, no significant difference.

**Figure S2.** **hMSCs promote tumor growth though activating MAPK signaling pathway in vivo.**

(a) Representative Western-bloting for Cyclin D1, pHH3 and pERK in tumors from xenograft transplantation model in the HCC-hMSCs group and HCC group. (b) Representative Western-bloting for Cyclin D1, pHH3 and pERK in tumors from orthotopic transplantation model in the two groups.

**Figure S3.** **Effects of hMSC on proliferation of HCC *in vitro*.**

(a, b) The effect of hMSCs on cell proliferation was analyzed by photographs. (c) The effect of hMSCs on cell cycles were analyzed by flow cytometry assay. (d) Different cell cycles were analyzed. (e) Proliferation associated genes PCNA, Cyclin D1, pHH3 and pERK were detected by western bloting assay between HCC-hMSCs group and HCC group. mean + SEM; NS, no significant difference.

**Figure S4.** **Top-listing upregulated and downregulated genes in RNA-seq results were confirmed by realtime PCR.**

(a) Some selected genes were significantly lower expression after hMSCs co-culture in RNA-seq. (b) Some selected genes were significantly higher expression after hMSC co-culture in RNA-seq. (c) The realtime PCR were used to confirm genes significantly lower expression after hMSCs co-culture in xenograft model. (d) The realtime PCR were used to confirm genes significantly high expression after hMSCs co-culture in xenograft model. (e) The realtime PCR were used to confirm genes significantly lower expression after hMSCs co-culture in vitro. (d) The realtime PCR were used to confirm genes significantly high expression after hMSCs co-culture in vitro.
